# The validity of the Strengths and Difficulties Questionnaire (SDQ) for children with ADHD symptoms

**DOI:** 10.1371/journal.pone.0218518

**Published:** 2019-06-19

**Authors:** Charlotte L. Hall, Boliang Guo, Althea Z. Valentine, Madeline J. Groom, David Daley, Kapil Sayal, Chris Hollis

**Affiliations:** 1 School of Medicine, Division of Psychiatry and Applied Psychology, Institute of Mental Health, University of Nottingham Innovation Park, Nottingham, United Kingdom; 2 University of Nottingham, CANDAL (Centre for ADHD and Neuro-developmental Disorders across the Lifespan) & MindTech, Developmental Psychiatry, University of Nottingham, Queen’s Medical Centre, Nottingham, United Kingdom; Vall d’Hebron University, SPAIN

## Abstract

**Background:**

The Strengths and Difficulties Questionnaire (SDQ) is widely used to assess child and adolescent mental health problems. However, the factor structure of the SDQ is subject to debate and there is limited evidence investigating measurement equivalence invariance (ME/I) between treatment groups, informants, and across time.

**Method:**

A randomised controlled trial (RCT) recruited 250 participants (6–17 years) who had been referred for an attention deficit hyperactivity disorder (ADHD) assessment. Participants and their clinician either received or did not receive a QbTest report (computer task measuring attention, impulsivity and activity). Parents and teachers completed the SDQ at baseline and 6-months later. This study aimed to understand the factor structure of the SDQ in a clinic referred ADHD sample, and validate the scale as a screening/diagnostic aide and as a measure of treatment outcome both in clinical and research settings. Exploratory Structural Equation Modelling (ESEM) was performed to examine the factor structure, and ME/I was assessed between treatment groups, informants, and time points. The criterion validity of the SDQ predictive algorithm for ADHD was compared with clinician and research diagnoses using logistic regression and tests of diagnostic accuracy.

**Results:**

A 5-factor structure provided the best fit with strong factorial invariance between treatment groups and across time points, but not across informants (parent and teacher ratings). SDQ ratings of ‘probable’ hyperactivity disorder were good predictors of clinical (OR = 10.20, 95%CI 2.18–48.71,p = 0.003) and research diagnoses of ADHD (OR = 6.82, 95%CI 1.95–23.84,p = 0.003), and research diagnoses of Hyperkinetic disorder (OR = 4.02, 95%CI 1.13–14.25,p = 0.031). Further examination of the SDQ hyperactivity ‘probable’ rating showed good specificity (84.5%-74.5%) but poor sensitivity (45.0–42.5%) for ADHD.

**Conclusion:**

The findings indicate the SDQ is a valid outcome measure for use in RCTs and clinical settings. However, care should be taken when using the SDQ predictive algorithm to screen for ADHD in clinically referred samples.

## Introduction

The Strengths and Difficulties Questionnaire (SDQ) is a brief, 25-item, measure of behavioural and emotional difficulties that can be used to assess mental health problems in children and young people aged 4–17 years [1, 2]. The SDQ can capture the perspective of the child/young person (self-report), or their parents and teachers perspective of the child’s symptoms. The self-report version of the SDQ can be completed by children and young people aged 11–17 years old, however, it is not advised for children younger than 11 years old. The parent and teacher versions can be completed by the parent or teacher of children and young people aged 4–17 years old [1]. The SDQ can be used as a screening tool, or to measure treatment outcome [3], and has been extensively implemented across countries for research and clinical purposes [4]. It is available in over 40 different languages and can be used without charge for non-commercial purposes [5]. As a frequently used clinical and research tool, the psychometric properties [2, 5, 6] and user-acceptability [7] of the SDQ have been well established. However, there has been limited or mixed evidence with regard to: 1) the factor structure of the SDQ in clinic-referred condition-specific populations [4]; 2) the measurement invariance between parent and teacher ratings [8]; 3) the predictive validity of the tool for specific disorders [9]; and 4) the validity of the SDQ as a longitudinal outcome measure in research trials. It is important to address these issues to inform clinicians about the different dimensions of psychopathology being measured through the SDQ and to aid the interpretation of results from randomised controlled trials (RCTs) and epidemiological studies.

The SDQ has five scales (scored 0–10); emotional problems, conduct problems, hyperactivity, peer problems and pro-social scale. The scales are combined (excluding the pro-social scale) into a ‘total difficulties’ score (0–40). Research on the factor structure of the SDQ has found inconsistent factor structures with the number of factors ranging from 3–5 [4, 10–16]. The proposed three factor structure includes two broad factors ‘internalising’ (consisting of the peer and emotional sub-scales) and ‘externalising’ (consisting of the conduct and hyperactivity sub-scales), with an additional pro-social factor. The five-factor structure consists of the five sub-scales.

The majority of studies exploring the factor structure of the SDQ have been conducted using general population samples [4, 10, 12, 14–16] with few studies examining its structure using clinical samples, despite the fact SDQ is used clinically to inform assessment, diagnosis and outcomes [4]. For example, Goodman et al. [4] conducted a large study in the general population and found support for the broader 3-factor model, however, discriminant validity between the scales was worse for children with low scores (indicating worse functioning), suggesting the 3-factor structure may not be best suited to a clinic-referred sample. The SDQ is frequently used as a clinical tool to aid the diagnostic assessment of attention deficit hyperactivity disorder (ADHD), a commonly occurring neurodevelopmental disorder [17], therefore it is particularly important to understand how best to use the SDQ in this population. In a large cross-European study, the psychometric properties of the parent version of the SDQ were investigated in a clinical sample of children with ADHD (aged 6–18 years). Factor analysis confirmed the 5-factor structure corresponding to the five scales. However, this study only used data from one informant. It is necessary to consider the factor structure of the SDQ for both teacher and parent informants [18] as the SDQ is typically rated by both sets of informants in clinical as well as research settings [13, 19] under the assumption that the ratings have the same operational meaning regardless of informant. However, there is often a difference between parent and teacher rating scores [20] which may be because the raters have observed the children in different environments and/or at different time points. As such, it is unclear whether these informant ratings are measuring the same underlying construct, which warrants further investigation.

Furthermore, there is a lack of research on the utility of the SDQ for predicting specific disorders such as ADHD. Across disorders, the SDQ has been shown to correctly identify 81–91% of children with a psychiatric diagnosis in a referred sample [9] and 63% of children in a community sample [21]. Of the limited research investigating the SDQ as a predictor of ADHD, one study conducted with a clinic-referred sample in Australia reported 44% sensitivity for hyperactivity disorders compared to clinical diagnosis [22]. Similarly, Rimvall et al. [23] noted 46% sensitivity for ADHD in a Danish community sample who were later diagnosed with ADHD. In another study, [21] reported 75% sensitivity to ADHD measured against Development and Wellbeing Assessment (DAWBA [3]) assigned diagnoses in a British community sample. There is also some uncertainty as to which informant is better: [21] noted that teacher SDQ scores were better than parent SDQs for predicting ADHD in a community sample, however, in another study there was no difference in parent and teacher completed SDQ scores as a predictor for receiving any psychiatric diagnosis in a clinical ‘looked after’ sample [24].

The SDQ has been frequently used in research trials to compare outcomes between two groups and across time points. It was recently used as a measure in a multi-centre RCT (AQUA-Trial) for children and young people referred to clinics for an assessment of attention deficit hyperactivity disorder (ADHD) [19, 25]. However, there is a lack of evidence investigating the stability of the factor structure of the SDQ across time points and groups.

It is important to compare SDQ scores between the experimental intervention and control groups such as in the AQUA-Trial to assess the measurement equivalence/invariance (ME/I) of the SDQ measures between groups so as to ensure the SDQ measures the same latent constructs in the same way. Previous evidence of the SDQ ME/I have been reported by gender, ethnicity groups, geographical regions, age and school year groups [4, 12, 14–16], but not across follow-up time and randomisation arms when the SDQ has been used as an outcome measure over time such as in a clinical trial.

Among studies investigating the factor structure of the SDQ, Exploratory Factor Analysis (EFA) and especially Confirmatory Factor Analysis (CFA), have been used as the analytical methods. In CFA modelling, each item is generally allowed to load on one factor and all non-target loadings are constrained to zero. In applied research, it is generally justifiable by theory and/or item contents that item(s) can cross-load on different latent factors [5, 10, 14]. Thus, restrictive zero loading typically results in an inflated CFA factor correlation and leads to biased estimates in CFA modelling when other variables are included in the model [26]. EFA is limited by its incapacity to incorporate latent EFA into the subsequent analysis, relating to other constructs or changes over time, nor does the approach lend itself to measure invariance across groups [26]. A recent methodological development–Exploratory Structural Equation Modelling (ESEM), integrates the advantages of both EFA and CFA. ESEM combines elements from EFA (e.g. allowing cross-loadings) to specify the underlying factor structure, together with applying the advanced statistical methods typically associated with CFAs (e.g. goodness-of-fit) [26]. To date, ESEM has not been performed to test the SDQ factor structure for clinically referred ADHD samples.

Given the SDQ is frequently used as a screening and outcome measurement tool for ADHD in clinic and research settings, there is a need to further understand the factor structure and the accuracy of the parent and teacher completed SDQ in detecting ADHD in a clinic-referred sample. This study primarily aimed to understand the factor structure of the SDQ in this sample, as well as validate the scale as a screening/diagnostic tool, and as a measure of treatment outcome both in clinical and research settings. Using ESEM and logistic regression, this study aimed to investigate, 1) the factor structure of the SDQ in a clinic sample of children referred for an ADHD assessment who participated in the AQUA-Trial, 2) the measurement invariance between parent and teacher informants, and 3) the measurement invariance between the two treatment groups of the AQUA-Trial across follow-up time points (baseline and six months) and 4) the diagnostic accuracy of the SDQ to detect ADHD in a clinic referred sample.

## Method and measures

### Participants

The sample were *n* = 250 children participating in the AQUA-Trial [19]. The AQUA-Trial was a two-arm, parallel group single-blind multi-centre diagnostic RCT conducted across 10 CAMHS/ community paediatric clinic sites in England. The aim of the trial was to investigate whether providing clinicians and families with an objective report of attention, impulsivity and activity (QbTest report) would accelerate diagnostic decision-making (both confirming and excluding ADHD) without compromising diagnostic accuracy [19, 25]. The trial was prospectively registered with ClinicalTrials.gov (NCT02209116; https://clinicaltrials.gov/ct2/show/NCT02209116), it was also later registered with the ISRCTN (ISRCTN11727351; https://www.isrctn.com/ISRCTN11727351). The QbTest is a computerised assessment of ADHD, comprising of a Continuous Performance Test with infrared camera to track motion during the test. The QbTest report presents the performance of an individual child against a norm-referenced database and indicates the severity of inattention, impulsivity and hyperactivity. Participants and their assessing clinician were randomised to either immediately receive the QbTest report (QbOpen group) or the report was withheld (QbBlind group). Both groups were followed for six-months from first appointment (baseline).

Eligible participants were children aged 6–17 years referred for their first ADHD assessment to a CAMHS or community paediatric clinic. Exclusion criteria were previous or current ADHD diagnosis or assessment, being non-fluent in English, and suspected moderate/severe intellectual disability. Informed consent was obtained from all individual participants included in the study. When the child was under 16-years-old, parents provided written consent for their child’s participation, verbal or written assent was also gained from the young person. When the child was 16-years-old or over, both the parent and young person provided written consent. Ethical approval was granted by Coventry and Warwick Research Ethics Committee (Ref: 14/WM/0166) and research and development (R&D) permissions were granted at each participating Trust. The research has been performed in accordance with the ethical standards as laid down in the 1964 Declaration of Helsinki and its later amendments. Outcome assessors were blind to group allocation throughout the trial. Details on the trial protocol and its primary outcome have been published [19, 25].

### Measures

#### Strength and Difficulties Questionnaire (SDQ)

The parent and teacher rated SDQ consists of 25-items that are rated on a 3-point Likert scale (not true, somewhat true, and certainly true), with a mixture of positive and negatively phrased items. The 25 items are designed to be divided between five sub-scales. Each of the five sub-scales comprises of five questions each. These five sub-scales and their items are listed below:

1. *Emotional Symptoms*—5 items = “complains of headache/stomach ache”; “Many worries, often seems worried”; “Often unhappy, down-hearted or tearful”; “Nervous or clingy in new situations, easily loses confidence”; and “Many fears, easily scared”.
2. *Conduct Problems*—5 items = “Often has temper tantrums or hot tempers”; “Generally obedient, usually does what adults request'”; “Often fights with other children or bullies them”; “Often lies or cheats”; and “Steals from home, school or elsewhere”.
3. *Hyperactivity-Inattention*—5 items = “Restless, overactive, cannot stay still for long”; “Constantly fidgeting or squirming”; “Easily distracted, concentration wanders”; “Thinks things out before acting”; and “Sees tasks through to the end, good attention span'”.
4. *Peer Problems*—5 items = “Rather solitary, tends to play alone”; “Has at least one good friend”; “Generally liked by other children”; “Picked on or bullied by other children”; and “Gets on better with adults than with other children”.
5. *Pro-Social Behaviour*—5 items = “Considerate of other people’s feelings”; “Shares readily with other children (treats, toys, pencils, etc.)”;”Helpful if someone is hurt, upset or feeling ill"; "Kind to younger children"; and "Often volunteers to help others (parents, teachers, other children”.

A total difficulties score is generated from the sum of the four sub-scales of emotion, conduct, hyperactivity-inattention and peer problems (20 items). The items for pro-social behaviours are not included in the total difficulties score [5]. Individual scores for the five items of each subscale (emotional symptoms, conduct problems, hyperactivity, peer problems, pro-social) were summed to provide a score for the corresponding subscale. Higher scores represent more problems with the exception of the pro-social behaviour sub-scale. The total difficulties score was generated by summing the scores for all the scales except the pro social scale, the resultant score can range from 0–40. The total score was not calculated if one of the component scores was missing.

The standard SDQ can be supplemented with a brief impact supplement which assesses the impact of the child’s difficulties in terms of distress, social impairment, burden and chronicity [27]. The SDQ was a secondary outcome measure in the AQUA-Trial and was used to assess behavioural symptoms at baseline (first appointment for ADHD assessment) and 6 months later. The parent and teacher 25-item SDQ with impact supplement was utilised for this purpose.

The SDQ diagnostic prediction algorithm generates ‘unlikely’, ‘possible’, or ‘probable’ ratings for conduct, emotional, hyperactivity and any psychiatric disorders (http://www.sdqinfo.com/c4.html) by collating information on symptoms and impact from multiple informants [9]. SDQs were valid to predict ADHD if all the required input variables (scores on conduct, hyperactivity, emotion and the impact score) needed for the predictive algorithm were present. If a required variable was missing, no diagnostic outcome was generated and the case was excluded from the analyses on diagnostic accuracy.

#### Psychiatric diagnosis

**Development and Well Being Assessment (DAWBA)** Children were assigned psychiatric diagnosis based on the Development and Well Being Assessment (DAWBA [3]). The DAWBA is a package of interviews, questionnaires and rating techniques completed by parents and teachers and designed to generate ICD-10 and DSM-IV / DSM-5 [28] psychiatric diagnoses for young people aged 5–17 years.

The DAWBA computer algorithm estimates the probability of having a psychiatric disorder in bands of <.1%, .5%, 3%, 15%, 50% and > 70% based on large community-based population studies [3], the top two levels have been shown to reliably indicate presence of a clinician-rated diagnosis and can be used as an alternative to clinician rated diagnoses in research studies [29]. The parent DAWBA can take between 20 minutes to 2 hours to complete depending on the complexity of symptoms, and the teacher version takes less than 30 minutes. The DAWBA’s were completed at baseline, either online or on the telephone with a researcher.

**Consultation pro forma** As part of the trial, clinicians were required to complete a short structured clinical record pro forma after each consultation. This pro forma documented information about the consultation including whether a confirmed diagnostic decision on ADHD had been reached. Clinicians could make a confirmed positive ADHD diagnosis, a confirmed excluded ADHD diagnosis, or not reach a diagnostic decision about ADHD within the six-month follow-up period. The clinician’s diagnosis was made in accordance to DSM-IV/V criteria. For the purpose of this study, analysis was only conducted on confirmed positive or confirmed excluded ADHD diagnoses.

### Data analysis

The factor structure of the SDQ was explored using ESEM [26]. With reference to existing studies on the factor structure of SDQ, factors ranging from three to seven were tested using baseline and six-month follow-up data separately; these factor structures were then explored using combined baseline and follow-up data. Measurement Invariance (ME/I) tests between treatment groups at/and across follow-up time points were conducted with ESEM using the best fitting model which also had the most meaningfully interpretable factor structure. The longitudinal ME/I test between baseline and follow-up for all participants as one group was firstly conducted by following consecutive modelling steps: configural invariance, metric invariance test (item factor loading invariance) and scalar invariance (item threshold invariance) test [30, 31]. The between arm measurement invariance at/across measurement times was then performed using the same testing order as for overall longitudinal measurement invariance. However, with each test step, the model with relevant parameters set to be equal between groups at each measurement time was tested first, followed by model testing the invariance between arms across the two measurement time points (i.e., parameters were set equal between groups and across time points). ME/I between rating sources were tested using the combined teacher and parent ratings data. All ESEM models were conducted using software Mplus 7.4 in its default setting [32]. Ordinal item score was analysed with the WLSMV estimator and missing values were automatically accounted for using the full-information maximum likelihood approach built into Mplus under the missing at random (MAR) assumption [33, 34].

To evaluate the ESEM model fit, Comparative Fit Index (CFI), non-normed fit index (NNFI) and Root Mean Square Error of Approximation (RMSEA) along with χ^2^ test were examined. It is suggested that models with CFI> 0.9, NNFI>0.9 and RMSEA<0.8 are accepted as a good fit [35]. Stepwise model comparisons were made between each adjacent nested model (e.g. from configural to loading). Model comparisons were generally evaluated by reference to the χ^2^ change test. However, the χ^2^ change test is influenced by sample size and data non-normality [30, 36] whereas the CFI change is independent of both model complexity and sample size and not correlated with the overall fit measurements. We therefore primarily judged model improvement on the CFI change [30, 37], with a change of less than 0.01 CFI indicating no difference between two model fittings [36]. As the WLSMV estimator was used to analyse ordinal items scores [32], the Mplus DIFFTEST function were used to conduct χ^2^ difference tests between the two nested models. For all modelling with the combined dataset, the same item measured at baseline and follow-up were correlated due as they were repeatedly measure [30]. Specific model elaboration is reported where needed in the results section.

To test the criteria-related validity of the SDQ, separate logistic regressions using STATA 14 were conducted to investigate whether the SDQ can predict ADHD/hyperkinetic diagnosis made by 1) independent research criteria for ADHD based on the DAWBA-derived diagnosis (DSM-IV/V), 2) independent research criteria for HKD based on the DAWBA-derived diagnosis ICD-10, and 3) clinician rated diagnosis of ADHD. In order to further explore the SDQ’s validity as a screening tool tests of diagnostic accuracy were conducted on the SDQ predictive algorithm for hyperactivity. For the purpose of analysis, the three resulting scores (possible, probable and unlikely) was reduced to two variables: ‘probable’ hyperactivity SDQ predictions were counted as positive for ADHD and ‘possible’ and ‘unlikely’ as negative for ADHD [9].

## Results

In total, 250 participants were consented, randomised and received the intervention (QbTest with the report either disclosed or withheld). Of these 123 were in the intervention arm (QbOpen) and 127 in the control arm (QbBlind). The participants’ background and demographic characteristics were similar between arms (see Table 1). Table 1 presents the baseline SDQ scores.

**Table 1:** Characteristics of the sample.

	QbBlind (control; report withheld) (*n* = 127)	QbOpen (intervention; report disclosed) (*n* = 123)	Total sample (*n* = 250)
**Sex (%)**			
Male	102 (80)	95 (77)	197 (79)
Female	25 (20)	28 (23)	53 (21)
**Age (years)**			
Mean age (SD)	9.4 (2.8)	9.5 (2.8)	9.5 (2.8)
Min-max	(5.9, 16.2)	(6.0, 17.4)	(5.9, 17.4)
Ethnicity (%)*	*n* = 89	*n* = 83	*n* = 172
White	80 (90)	73 (88)	153 (89)
Mixed	5 (6)	6 (7)	11 (6)
Other	(4 (4)	4 (5)	8 (5)
SDQ Parent (SDQ-P)*: Mean (SD)	*n* = 108	*n* = 90	*n* = 198
Emotional problems	4.9 (2.8)	4.4 (2.9)	4.7 (2.9)
Conduct problems	5.9 (2.4)+	5.9 (2.7)+	5.9 (2.5)
Hyperactivity	8.8 (1.3)++	8.9 (1.6)++	8.9 (1.4)
Peer problems	4.6 (2.4)+	4.1 (2.4)+	4.4 (2.4)
Pro-social behaviour	5.3 (2.3)	5.6 (2.1)	5.5 (2.3)
Total difficulties score	24.3 (5.9)+	23.3 (6.2)+	23.9 (6.1)
Impact score	5.9 (2.6)+	5.8 (2.6)+	5.9 (2.6)
SDQ Teacher (SDQ-T)*:Mean(SD)	*n* = 85	*n* = 75	*n* = 160
Emotional problems	2.9 (3.1)	2.7 (2.6)	2.8 (2.9)
Conduct problems	3.9 (2.9)	3.3 (2.7)	3.6 (2.9)
Hyperactivity	7.6 (2.5)+	7.2 (2.8)+	7.4 (2.6)
Peer problems	2.9 (2.3)	2.4 (2.8)	2.7 (2.3)
Pro-social behaviour	5.2 (2.4)	5.3 (2.5)	5.2 (2.4)
Total difficulties score	17.5 (7.4)+	15.7 (6.9)	16.6 (7.2)
Impact score	3.0 (2.0)+	2.6 (1.7)+	2.8 (1.9)
**Type of clinical service** (%)	*n* = 127	*n* = 123	*n* = 250
CAMHS	60 (47)	59 (48)	119 (48)
Community Paediatrics	67 (53)	64 (52)	131 (52)

*Note*. Data are n (%) or mean (SD/range). ‘Other’ ethnicity includes Pakistani, Indian and Other Asian.

*Data not available for all randomised participants.

^+^ scores are in the abnormal range (top 10%)

^++^ scores are in the top 5%.

CAMHS = child and adolescent mental health services. Higher scores on the Strengths and Difficulties Questionnaire (SDQ) indicate more problems with the exception of pro-social behaviour. SDQ scores are at baseline.

### SDQ factor structure

In order to test the proposed factor structures, modelling fitting results with factor structures ranging from 3–7 for baseline and follow-up data were obtained (see S1 and S2 Tables for parent and teacher data). S1 and S2 Tables present the data for baseline and follow-up separately. The results showed that the models with more factor numbers generally fitted the data better. However, CFIs did not make any substantial improvements with models of 6 and 7 factors against a model with 5 factors among three out of four datasets, indicating a 5-factor model is the best fit. This was confirmed by the model fitting results of a configural invariance model conducted on combined baseline and follow-up data combined in the same model, participants were included in this analysis if they had at least one completed SDQ measure (at baseline or follow-up). The results showed that 6 and 7 factors did not fit the data any better than a 5-factor model, with CFI gains of less than 0.01 (Table 2). Given the substantial CFI gains with more factors, the results do not support the broader 3-factor internalising and externalising factors.

**Table 2:** Fittings indices of configural invariance ESEM models with different factors (parent and teacher data).

Model	χ^2^(df), p =	RMSEA	CFI	NNFI	Δχ^2^(Δdf), p =	ΔCFI
**Parent data (n = 216)**						
3-factor	1276.011(1047),0.000	.032	.941	.931		
4-factor	1157.900(996), 0.000	.027	.958	.949	120.703(51),0.000	0.017
5-factor	1036.759(945), 0.020	.021	.976	.969	115.412(51),0.000	0.018
6-factor	954.825(894), 0.077	.018	.984	.979	87.032(51),0.001	0.008
7-factor	875.184(843), 0.215	.013	.992	.988	84.214(51),0.002	0.008
**Teacher data (n = 189)**						
3-factor	1293.662(1047),0.000	.035	.954	.946		
4-factor	1165.792(996), 0.000	.030	.968	.961	126.787(51),0.000	.014
5-factor	1037.104(945), 0.019	.023	.983	.978	120.284(51),0.000	.015
6-factor	959.046(894), 0.065	.020	.988	.983	79.623(51), 0.006	.005
7-factor	878.693(843), 0.191	.015	.993	.990	84.144(51), 0.002	.005

*Note*. RMSEA = root mean square error of approximation; CFI = comparative fit index; NNFI = non-normal fit index. N = number of participants with at least one completed baseline or follow-up measure

**Fig 1:**
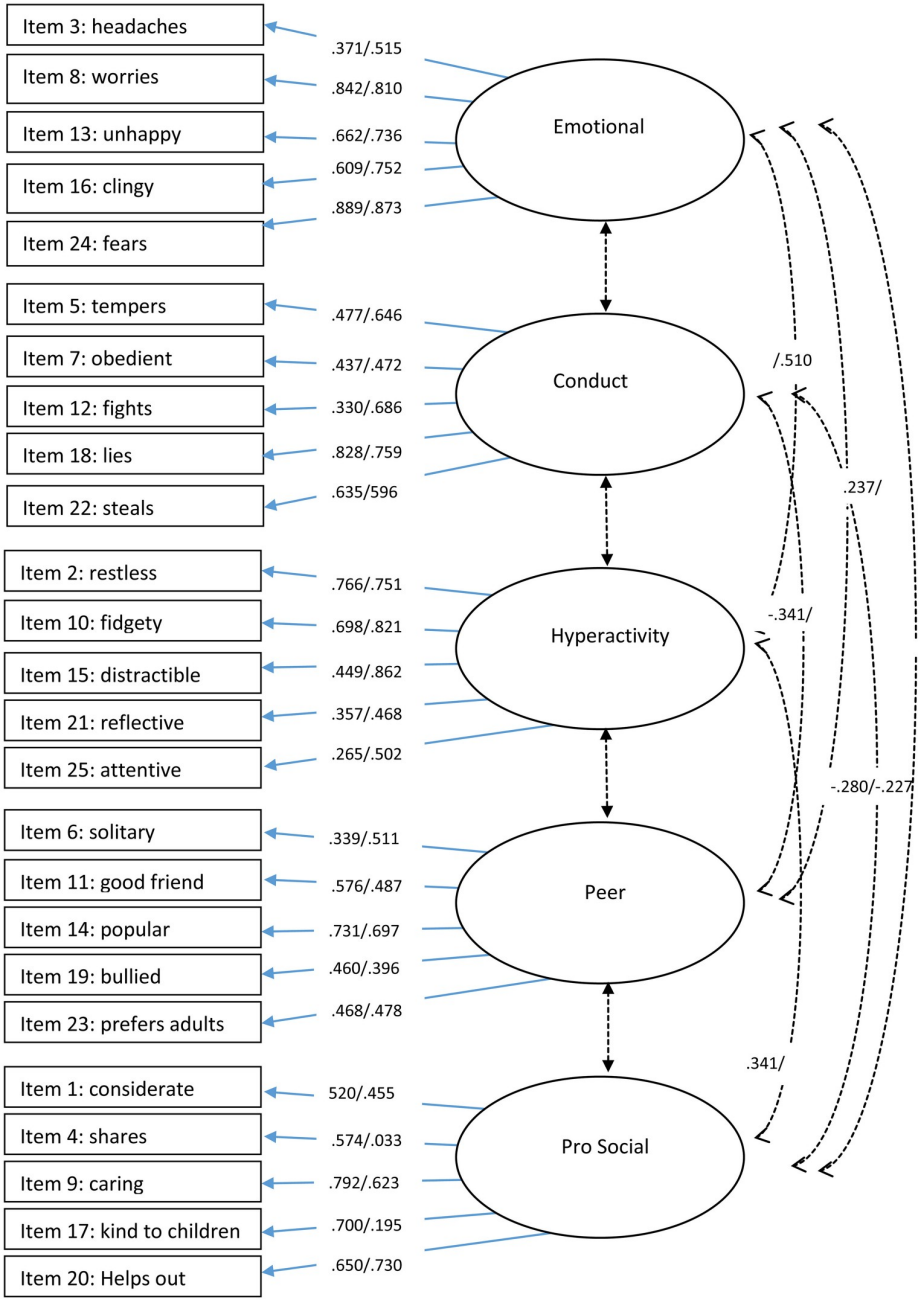
Item factor loadings and correlations for baseline/follow-up parent data. *Note*. Only significant correlations are presented. Cross item loadings are presented in S1 Table. Data is shown as baseline/follow-up.

**Fig 2:**
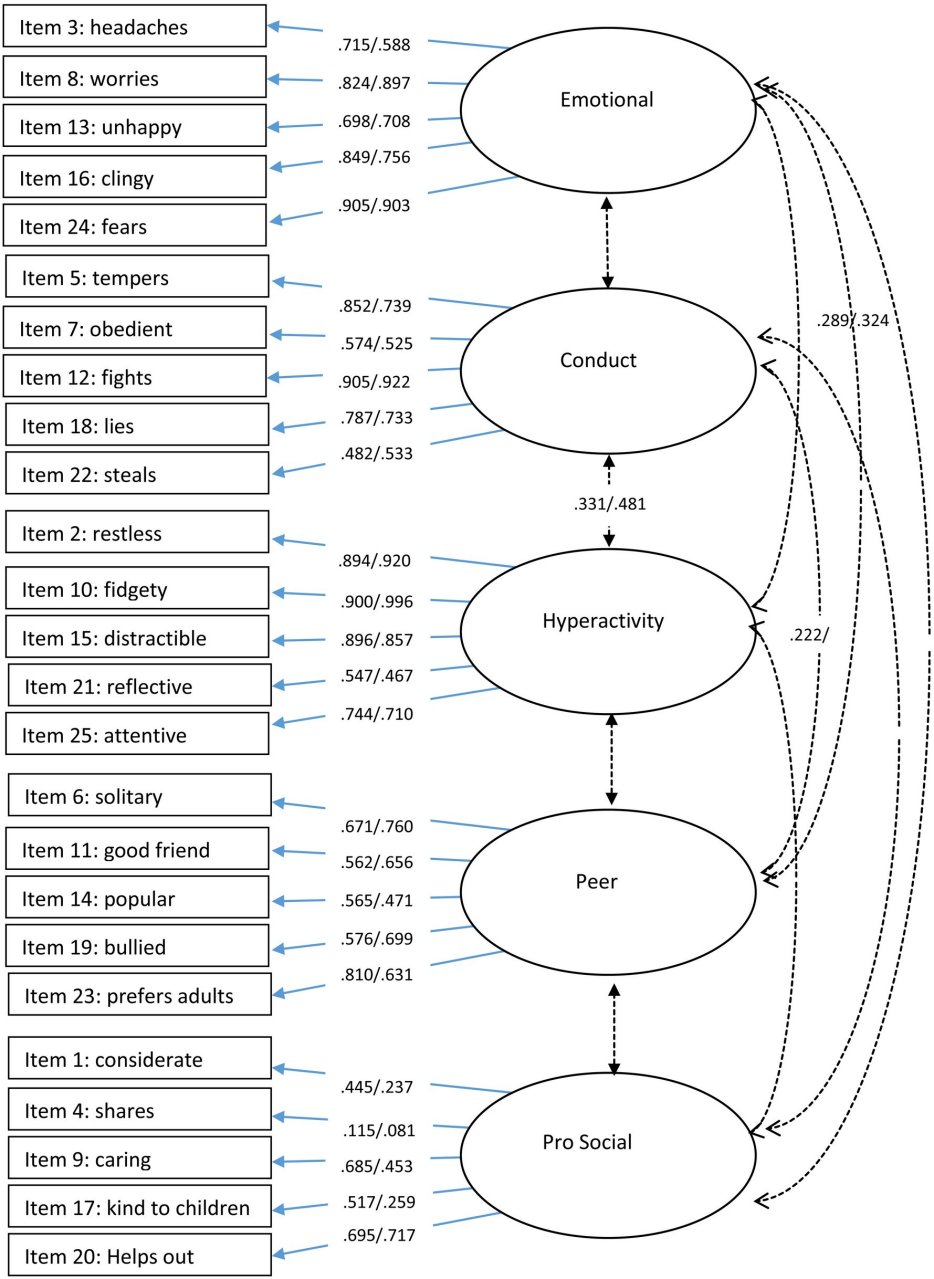
Item factor loadings and correlations for baseline/follow-up teacher data. *Note*. Only significant correlations are presented. Cross item loadings are presented in S2 Table. Data is shown as baseline/follow-up.

To further explore this, we investigated the factor loading pattern of the different structures. Meaningful loadings were assessed using the criteria of 0.32 ("poor"), 0.45 ("fair"), 0.55 ("good"), 0.63 ("very good"), and 0.71 ("excellent") [38]. The loading pattern confirmed that the 5-factor structure was the best-fitting model. The factor loadings and item factor mapping from the 5-factor model using baseline and follow-up data are presented in Fig 1 (parent data) and Fig 2 (teacher data) (See also S3 and S4 Tables). For parent data, items from the emotional factor generally showed good-to-excellent fit. Items from the conduct and hyperactivity and peer factor generally loaded fair-to-excellent. Items from the pro-social factor also loaded fair-to-excellent, however, items 4 (‘shares’) and 17 (‘kind to children’) did not significantly load on to this factor at the follow-up time point.

For teacher data (Fig 2), items from emotional, conduct, hyperactivity and peer factors generally showed good-to-excellent fit. Items from the pro-social factors loaded from poor-to-excellent, again, with items 4 (‘shares’) and 17 (‘kind to children’) not significantly loading on to this factor at either one or both follow-up time points.

Factor loadings for individual items show that some items had large cross-loadings. Although most items loaded onto the primary factor above 0.40, several items also loaded onto alternative factors greater than 0.30, and/or there was not a difference of 0.20 between factor loadings. This was true for 7 items for the parent (see S3 Table) and 7 items for the teacher data (see S4 Table). For parent data, the greatest cross-factor item loadings were with the ‘emotion’ factor. For teacher data, the largest cross-factor item loadings were with the ‘conduct’ factor.

Significant correlations between factors are presented in Fig 1 (parent data) and Fig 2 (teacher data). For parent data, weak negative correlations were found between the pro-social factor and peer and conduct factor, a stronger negative correlation between the conduct and pro-social factor was also found with teacher data (.427). The strongest correlation was found between hyperactivity and emotion for parent data at the follow-up time point (.510).

### Measurement invariance test of a 5-factor structure across time points

For the ESEM modelling, we collated data from both treatment groups and sequentially ran ME/I test models of: configural invariance, loading invariance and item threshold invariance. The model fit indices for each ME/I model are presented in Table 3. The threshold invariance model results showed that the 5-factor structure model evidenced a strong factorial invariance across measurement time points (baseline and follow-up).

**Table 3:** Model fit indices of longitudinal ME/I modelling.

Model	χ^2^(df), p =	RMSEA	CFI	NNFI	Δχ^2^(Δdf), p =	ΔCFI
**Parent data (n = 216)**						
Configural	1036.759(945), 0.020	.021	.976	.969		
Loading	1162.567(1045),0.06	.023	.970	.964	86.792(126),0.997	.006
Threshold	1288.091(1095),0.000	.029	.950	.944	212.387(50),0.000	-.020
Threshold*^1^	1235.593(1088),0.001	.026	.962	.957	111.632(43),0.000	-.008
**Teacher data (n = 189)**						
Configural	1037.104(945), 0.019	.023	.983	.978		
Loading	1109.484(1045),0.081	.018	.988	.986	100.912(100),0.456	.005
Threshold	1241.459(1095),0.001	.027	.973	.969	191.334(50),0.000	.015
Threshold*^2^	1232.742(1093),0.002	.026	.974	.971	179.478(48),0.000	.001

Note.

*^1^ free 7 threshold estimates between follow-up time.

*^2^ Free 2 threshold estimates between follow-up time.

RMSEA = root mean square error of approximation; CFI = comparative fit index; NNFI = non-normal fit index. N = number of participants with at least one completed baseline or follow-up measure

Next, we tested the longitudinal measurement invariance between the two treatment groups across time points for parent and teacher data. The various ME/I model fitting data for parent and teacher data are presented in Table 4. Again, the results showed strong factorial invariance, indicating the 5-factor structure model with similar loading patterns remained stable between baseline and follow-up for parent data, with a small amount of item threshold estimates freely estimated between baseline and follow-up time.

**Table 4:** Model fit indices of ME/I modelling between arms at/cross follow-up time and between informant.

Model		χ^2^(df), p =	RMSEA	CFI	NNFI	Δχ^2^(Δdf), p =	ΔCFI
**Parent (n = 216)**							
Configural		1995.014(1890),0.046	.023	.972	.963		
Loading	A	2205.691(2090),0.039	.023	.969	.963	244.710(200),0.017	.003
	B	2307.329(2190),0.040	.022	.968	.965	118.695(100),0.098	.001
Threshold	A	2397.649(2289),0.056	.021	.971	.969	89.713(99),0.737	-.003
	B*^1^	2467.048 (2334),0.027	.023	.964	.962	136.137(45),0.000	.007
**Teacher (n = 189)**							
Configural		2015.748(1890),0.022	.027	.975	.968		
Loading	A	2227.980(2090),0.018	.026	.973	.968	254.259(200),0.006	-.002
	B	2325.709(2190),0.022	.026	.973	.970	120.286(100),0.082	.000
Threshold	A	2414.825(2290),0.034	.024	.975	.974	90.047(100),0.752	.002
	B*^2^	2509.498(2338),0.007	.028	.966	.964	177.368(48),0.000	-.009
**ME/I test between informant at/cross follow-up time (n = 237)**							
Loading	C	4716.044(4490),0.009	.015	.964	.960	278.513(200),0.000	-.009
	D	4820.930(4590),0.009	.015	.963	.960	128.646(100),0.028	-.001
Threshold	C	5272.245(4690),0.000	.023	.907	.902	1067.617(100),0.000	-.058
	C*^3^	5127.319(4678),0.000	.020	.928	.924	709.914(88),0.000	-.035

*Note*. A: equal parameters between arms at each time, B: equal parameters between arms across follow-up time.

*^1^ free 4 item threshold parameter estimates between baseline and follow up time.

*^2^ Free estimates of the 1st threshold of item 18 and the 1st threshold of item 25 between times. C: invariant between informants, D: invariant between informants across time,

*^3^ free 10 largest threshold parameter estimates between baseline and follow-up time.

RMSEA = root mean square error of approximation; CFI = comparative fit index; NNFI = non-normal fit index. N = number of participants with at least one completed baseline or follow-up measure

The longitudinal measurement invariance between parent and teacher data were then compared across time points. The results are presented in Table 4 and show strong factorial invariance for the 5-factor structure, as the invariant threshold model was not tenable (model threshold C in Table 4). The model fitted the data well but the CFI dropped 0.058 (i.e., more than 0.01 cut-off value) from invariant loading model, even using a partial invariant threshold model freeing 12 threshold estimates between the parent and teacher data still dropped the CFI to 0.035 from the equal loading model (model threshold C*). Thus, invariant threshold estimates between parent and teacher ratings were not evidenced.

### Association between SDQ algorithm and ADHD diagnosis

To test the criterion validity of the SDQ, we investigated the association between the SDQ diagnostic algorithm and the child’s diagnosis assigned by the clinician and the DAWBA. The SDQ algorithm predicted that a hyperactivity disorder was probable in 35% (79/228), possible in 59% (135/228) and unlikely in 6% (14/228) of the sample. Table 5 shows the association between SDQ predictions and independent DSM-IV/V and ICD-10 research diagnosis from DAWBA predictions and clinician assigned diagnoses (DSM-IV). For each diagnostic criteria (DAWBA ICD-10, DAWBA-DSM- IV/V, clinician diagnosis), children with an SDQ prediction of ‘probable’ were more likely to receive an ADHD/hyperkinetic diagnosis than have ADHD/hyperkinetic disorder excluded but there were no significant effects for ‘possible’ ratings.

**Table 5:** Association between Strengths and Difficulties Questionnaire (SDQ) prediction and psychiatric diagnosis from DAWBA and clinician for ADHD/hyperkinetic disorder.

SDQ prediction	DAWBA ICD-10 HKD present *N* = 221			DAWBA DSM-IV/V ADHD present *N* = 222			Clinical diagnosis ADHD present*N* = 152		
	No n(%)	Yes n(%)	OR(95%CI),p =	No n(%)	Yes n(%)	OR(95%CI),p =	No n(%)	Yes n(%)	OR(95%CI),p =
**Unlikely**	9 (4.1)	4 (1.8)		8(3.6)	5 (2.3)		9(3.9)	5(2.2)	
**Possible**	73 (33.0)	57 (25.8)	1.76(0.51,6.00), 0.368	51(23.0)	79 (35.6)	2.48(0.77,8.00),0.129	71 (31.4)	64 (28.1)	2.9(0.77,11.01),0.116
**Probable**	28 (12.7)	50 (22.6)	4.02(1.13,14.25), 0.031	15(6.8)	64 (28.9)	6.82 (1.95,23.84),0.003	28 (12.3)	51 (23.7)	10.2 (2.18,47.71),0.003

*Note*. Clinical diagnosis represents the diagnosis recorded on the clinical pro forma. Only definitive ‘ADHD present’ or ‘ADHD not present’ diagnoses were included in the analysis. CI = confidence interval. HKD = hyperkinetic disorder. OR = Odds Ratio.

The sensitivity/specificity and positive/negative predictive value of the SDQ ‘probable’ ratings against clinician and independent research diagnoses (DAWBA) are presented in Table 6 and show the SDQ algorithm is not sensitive to detecting ADHD but is reasonable at identifying patients without ADHD.

**Table 6:** The diagnostic accuracy of the SDQ ‘probable’ algorithm.

	DAWBA ICD-10 HKD present *N* = 221	DSM-IV/V ADHD present *N* = 222	Clinical diagnosis ADHD present *N* = 152
**Sensitivity % (95%CI)**	45.0(35.6, 54.8)	43.2(35.1, 51.6)	42.5(33.5, 51.9)
**Specificity % (95%CI)**	74.5(65.4, 82.4)	79.7(68.8, 88.2)	84.4(64.2, 94.7)
**Positive Predictive Value % (95%CI)**	64.1(52.4, 74.7)	81.0(70.6, 89.0)	91.1(80.4, 97.0)
**Negative Predictive Value % (95%CI)**	57.3(48.8, 65.6)	41.3(33.1, 49.8)	28.1(19.4, 38.2)

Note. CI = confidence interval. DAWBA = Development and Well Being Assessment. HKD = hyperkinetic disorder.

## Discussion

Given that the SDQ is internationally a widely used clinical and research tool to aid the diagnostic assessment of ADHD we aimed to investigate the factor structure and the accuracy of the SDQ in detecting ADHD in a clinic-referred sample using novel and vigorous ESEM techniques, alongside logistic regression and tests of diagnostic accuracy. The findings revealed that a 5-factor structure (emotional problems, conduct problems, hyperactivity problems, peer problems and pro-social behaviour) for both parent and teacher data was the best fit. This 5-factor structure showed strong factorial invariance across time points (baseline and follow up) indicating the validity of the 5-factor structure as an outcome measure. However, strong measurement invariance was not evidenced between parent and teacher scores, indicating parents and teacher data measure the same construct but in slightly different way. To our knowledge this is the first time cross-time measurement invariance or treatment group invariance, and informant (parent/teacher) invariance across time has been investigated for the SDQ. Furthermore, we showed that scores on the SDQ hyperactivity scale were associated with a research and clinical diagnosis of ADHD in a referred sample. However, further analysis on sensitivity/specificity demonstrated that the SDQ predictive algorithm was not sensitive to ADHD.

Model fitting information comparing models with 3–7 factors showed that the 5-factor model remained superior over time for both parent and teacher data. Uniquely, this is the first study to show that this 5-factor model demonstrated strong factorial measurement invariance across time points (baseline and follow-up) and between treatment groups (QbOpen and QbBlind) across time points. In addition, generally poor correlations were found between the five factors suggesting that the factors are broadly measuring independent concepts. With regard to evidence for broader ‘internalising’ and ‘externalising’ factors, poor (but positive) correlations were found between the emotional and peer-problem factors (proposed internalising factors) for both parent and teacher data (correlations ranged from .237-.324), and poor-to-fair correlations between conduct and hyperactivity (proposed externalising factors) were found for teacher data (correlations ranged from .331–4.81). However, the weak nature of these correlations combined with model fitting data suggest that a 3-factor model does not best fit the data. The confirmation of a 5-factor structure for the SDQ is consistent with findings from some studies using community samples [12–15] and supports the findings of Goodman et al. [4] that maintaining a 5-factor structure may be of particular value in a high-risk sample.

Although our findings evidenced a 5-factor structure consistent with the factors proposed by Goodman [5], some items showed cross-loadings with other factors. For parent data, the greatest cross-factor loadings were with the ‘emotion’ factor. Similar to the findings of Hawes and Dadds [39] and Goodman [5], respectively, the peer problem items ‘rather solitary’ and ‘picked on/bullied’ mapped onto the emotional factor, in our study a third peer problem item ‘gets on better with adults’ also loaded onto the emotion factor. Furthermore, the strongest correlation between factors was found with the hyperactivity and emotion factors. These findings indicate that in a sample of children referred over the question of possible ADHD there is likely to be a strong association between emotional problems and peer relationship problems including social isolation and peer bullying/ victimisation. Likewise, for parent data, a cross-factor loading for the conduct item ‘often has tempers’ was found with the emotion factor, supporting the findings of [15] and [40], again indicating an interplay between emotional and conduct problems in referred children. This was supported by teacher data which showed the emotional item ‘often unhappy’ loaded onto the conduct factor. Finally, our results showed that the two hyperactivity items ‘restless’ and ‘constantly fidgeting’ positively cross-loaded onto the pro-social factor for parent data and teacher data. Hawes and Dadds [39] also noted a loading of some hyperactivity items on the pro-social factor, suggesting that some social strengths may be associated with hyperactivity.

For teacher data, several other items cross-loaded onto the conduct scale, including the hyperactivity item ‘reflective’, the peer problems items ‘popular’ and ‘bullied’. Although the item ‘generally liked’ positively associated with conduct problems, it is important to note that the direction of scoring of this question is reversed so that scoring “very true” on this item would be a score of 0 to represent no/few issues with peer problems i.e. a positive association between peer relationship problems and conduct problems.

Given that ADHD is a highly heterogeneous condition, with individuals showing different degrees of symptom severity and impairment, as well as different patterns of symptoms, there is a need to better understand the diversity and commonality between symptoms to define more homogenous ADHD phenotypes [41].

The finding that positive (but weak) correlations between the internalising factors of peer problems and emotional problems as well as various cross loadings for items of hyperactivity, conduct, emotion and pro-social and peer problems suggest that although each are distinct symptom domains in ADHD, they are not fully separable, suggesting some convergence on the casual pathways [41]. The cross-loadings imply that difficulties in these symptom domains are likely to co-occur which may explain the heterogeneity in ADHD and supports previous research demonstrating neuropsychological heterogeneity in ADHD [42]. Further understanding of how these factors may interplay with the recognised sub-types of predominately inattentive or predominately hyperactive/impulsive may improve our understanding of ADHD as a heterogeneous condition. Our findings suggest that factor models of ADHD need to account for both concurrent overlap and separability between the ADHD symptoms domains, which requires further research.

Measurement invariance between treatment groups across time periods is necessary to be able to meaningfully compare outcomes between two treatment groups. The strong factorial invariance found between the two treatment groups at and across measurement time points and the weak factorial invariance between teacher and parents ratings, indicate that comparing the observed score between treatment groups (i.e. two trial arms) and time-points is valid. The untenable invariant threshold model between teacher and parents indicated there are systematic response differences towards the same child’s behaviour between teachers and parents [30], indicating the two informants results are not directly comparable. This may in part reflect that teachers and parents observe the child in different settings. This is also reflected in previous research which shows poor/moderate correlations between parent and teacher SDQ scores, for example, [43] found an average correlation of only 0.39 between parent and teacher scores in a clinic sample.

In contrast to previous studies, our findings showed mixed support for the utility of the SDQ to predict ADHD [9, 21, 24, 27]. Although participants with a ‘probable’ rating for hyperactivity on the SDQ algorithm were more likely to receive a clinical and research diagnosis of ADHD, further analysis on ‘probable’ scores showed low sensitivity but good specificity to ADHD, indicating that care should be taken when using the tool to aid in the screening for ADHD. However, it should be noted that our analysis was conducted using the SDQ algorithm (http://www.sdqinfo.com/c4.html) but without the third informant (self-report data), as such this may have weakened the predictive validity of our results and greater predictive validity may be found with a complete informant data set. This is perhaps particularly important for ADHD where self-report data is important. Furthermore, given the complexity of ADHD, the condition should never be diagnosed based on the result of one assessment tool [17].

Our findings are strengthened by the use of the novel ESEM approach, which combines both the benefits of EFA and CFA [26,44]. A limitation to our research is we did not collect self-report SDQ data, as such, future research may wish to conduct ESEM analysis comparing informants at different time points including SDQs completed by the young person. Furthermore, the analysis was conducted using data from participants in a RCT who had been referred for an ADHD assessment, although this provides the opportunity to understand the validity of the SDQ in this very frequently seen group in child mental health/paediatric services, the results may not apply to patients with other presenting symptoms/difficulties. Additionally, there may be differences in patients who agree to participate in a RCT compared with those who do not, and the results may be a reflection of the predominately white, British sample. Alongside this, although 250 children participated in the RCT, there was missing data for both parent and teacher SDQs as well as DAWBAs and clinician rated diagnoses. Given that participants were recruited from clinical settings this is not unexpected, as parents main motivation was likely to be to receive support for their child rather than fill in questionnaires, similarly as in other studies, teachers were not allocated teaching time to complete the questionnaires [45]. The attrition rate was in line with other research in clinical samples [45,46] and was fully accounted for in our analysis. The missing outcomes pattern were explored with no obvious evidence against the MAR assumption made on our data when applying the Full Information Maximum Likelihood (FIML) algorithm to handle missingness.

In conclusion, results of an ESEM approach showed that a 5-factor structure best fitted parent and teacher rated SDQs for a sample of children and young people referred to specialist services for an ADHD assessment. The 5-factor structure showed strong factorial measurement invariance across treatment groups and time points. Therefore, the factor structure of the SDQ should be considered a valid and robust outcome measure for future research studies and to inform clinical judgement of patient symptoms/improvement. Strong measurement invariance was not observed between parent and teacher ratings indicating differences in parent and teachers rating scores. Although ‘probable’ scores on the SDQ hyperactivity scale were a good predictor of receiving a clinical and research diagnosis of ADHD, the poor sensitivity of these scores for ADHD indicate that care should be taken when using the SDQ predictive algorithm to screen for ADHD.

## Supporting information

S1 Table ESEM model fitting indices across both baseline and follow-up (parent data). (DOCX)

S2 Table ESEM model fitting indices across both baseline and follow-up (teacher data). (DOCX)

S3 Table Five-factor item mapping and factor loading of 5-factor configural invariance (baseline/follow-up) esem model (parents rating). (DOCX)

S4 Table Five-factor item mapping and factor loading of 5-factor configural invariance (baseline/follow-up) esem model (teacher rating). (DOCX)
