# Supplementary material for: The validity of the Strengths and Difficulties Questionnaire (SDQ) for children with ADHD symptoms
Source: PLoS One. 2019 Jun 19;14(6):e0218518. doi: 10.1371/journal.pone.0218518 (PMC6583960; doi:10.1371/journal.pone.0218518)
Supplement: S1 Table — (DOCX) [file pone.0218518.s001.docx]

**S1. ESEM Model Fitting Indices across Both Baseline and Follow-up (Parent Data)**

| **Data** | **Factor** | **χ^2^(df), p =** | **RMSEA** | **CFI** | **NNFI** | **Δχ^2^(Δdf), p=** | **ΔCFI** |
| --- | --- | --- | --- | --- | --- | --- | --- |
| **Baseline**  **(n=198)** | 3 | 325.299(228),0.000 | .046 | .938 | .919 |  |  |
|  | 4 | 251.019(206),0.018 | .033 | .971 | .958 | 67.983(22),0.000 | .033 |
|  | 5 | 197.257(185),0.255 | .018 | .992 | .987 | 49.775(21),0.000 | .021 |
|  | 6 | 166.548(165),0.452 | .007 | .999 | .998 | 29.837(20),0.073 | .007 |
|  | 7 | 136.505(146),0.702 | .000 | 1.000 | 1.012 | 27.295(19),0.098 | .000 |
| **Follow**  **up**  **(n=163)** | 3 | 369.959(228),0.000 | .062 | .922 | .897 |  |  |
|  | 4 | 304.886(206),0.000 | .054 | .945 | .920 | 66.187(22),0.000 | .023 |
|  | 5 | 250.032(185),0.001 | .046 | .964 | .942 | 51.799(21),0.000 | .019 |
|  | 6 | 201.588(165),0.028 | .037 | .980 | .963 | 47.908(20),0.000 | .016 |
|  | 7 | 178.180(146),0.036 | .037 | .982 | .963 | 26.138(19),0.126 | .002 |

*^Note.^* ^RMSEA=root mean square error of approximation; CFI= comparative fit index; NNFI=non-normal fit index.^
