# Supplementary material for: The validity of the Strengths and Difficulties Questionnaire (SDQ) for children with ADHD symptoms
Source: PLoS One. 2019 Jun 19;14(6):e0218518. doi: 10.1371/journal.pone.0218518 (PMC6583960; doi:10.1371/journal.pone.0218518)
Supplement: S2 Table — (DOCX) [file pone.0218518.s002.docx]

**S2. ESEM Model Fitting Indices across Both Baseline and Follow-up (Teacher Data)**

| **Data** | **Factors** | **χ2(df), p =** | **RMSEA** | **CFI** | **NNFI** | **Δχ2(Δdf), p=** | **ΔCFI** |
| --- | --- | --- | --- | --- | --- | --- | --- |
| **Baseline**  **(n=160)** | 3 | 469.506(228),0.000 | .081 | .915 | .888 |  |  |
|  | 4 | 351.020(206),0.000 | .066 | .949 | .926 | 100.169(22),0.000 | .034 |
|  | 5 | 243.284(185),0.003 | .044 | .979 | .967 | 84.642(21),0.000 | .010 |
|  | 6 | 194.795(165),0.056 | .034 | .989 | .981 | 46.428(20),0.001 | .005 |
|  | 7 | 162.087(146),0.172 | .026 | .994 | .988 | 31.797(19),0.033 | .005 |
| **Follow**  **up**  **(n=154)** | 3 | 346.283(228),0.000 | .058 | .972 | .963 |  |  |
|  | 4 | 275.390(206),0.001 | .047 | .983 | .976 | 61.848(22),0.000 | .011 |
|  | 5 | 214.528185(),0.068 | .032 | .993 | .988 | 55.163(21),0.000 | .010 |
|  | 6 | 171.982(165),0.339 | .017 | .998 | .997 | 39.081(20),0.007 | .005 |
|  | 7 | 162.087(146),0.172 | .026 | .994 | .988 | 16.704(19),0.610 | -.004 |

*^Note.^* ^RMSEA=root mean square error of approximation; CFI= comparative fit index; NNFI=non-normal fit index.^
