# Supplementary material for: The validity of the Strengths and Difficulties Questionnaire (SDQ) for children with ADHD symptoms
Source: PLoS One. 2019 Jun 19;14(6):e0218518. doi: 10.1371/journal.pone.0218518 (PMC6583960; doi:10.1371/journal.pone.0218518)
Supplement: S3 Table — (DOCX) [file pone.0218518.s003.docx]

**S3. Five-Factor Item Mapping and Factor Loading of 5-Factor Configural Invariance (Baseline/Follow-up) ESEM Model (Parents Rating)**

|  | **Factors** | | | | |
| --- | --- | --- | --- | --- | --- |
| **Item** | **‘emotional’** | **‘conduct’** | **‘hyperactivity’** | **‘peer’** | **‘prosocial’** |
| 3 Often complained of headaches, stomach-aches or sickness | **.371/.515** |  |  |  |  |
| 8 Many worries, often seems worried | **.842/.810** |  |  |  |  |
| 13 Often unhappy, down-hearted or tearful | **.662/.736** |  |  | .360/ |  |
| 16 Nervous or clingy in new situations, easily loses confidence | **.609/.752** |  |  |  |  |
| 24 Many fears, easily scared | **.889/.873** |  |  |  |  |
| 5 Often has temper tantrums or hot tempers | .445/.424 | **.477/.646** |  |  |  |
| 7 Generally obedient, usually does what adults request * |  | **.437/.472** |  |  |  |
| 12 Often fights with other children or bullies them |  | **.330/.686** |  |  |  |
| 18 Often lies or cheats |  | **.828/.759** |  |  |  |
| 22 Steals from home, school or elsewhere |  | **.635/596** |  |  |  |
| 2 Restless, overactive, cannot stay still for long |  |  | **.766/.751** |  | /.570 |
| 10 Constantly fidgeting or squirming |  |  | **.698/.821** |  | /.464 |
| 15 Easily distracted, concentration wanders |  |  | **.449/.862** |  |  |
| 21 Thinks things out before acting * |  |  | **.357/.468** |  |  |
| 25 Sees tasks through to the end, good attention span * |  |  | **.265/.502** |  |  |
| 6 Rather solitary, tends to play alone | .405/.466 |  |  | **.339/.511** |  |
| 11 Has at least one good friend * |  |  |  | **.576/.487** |  |
| 14 Generally liked by other children * |  |  |  | **.731/.697** |  |
| 19 Picked on or bullied by other children | .261/.457 |  |  | **.460/.396** |  |
| 23 Gets on better with adults than with other children | .318/.308 |  |  | **.468/.478** |  |
| 1 Considerate of other people’s feelings |  |  |  |  | **.520/.455** |
| 4 Shares readily with other children (treats, toys, pencils etc.) |  |  |  |  | **.574/.033** |
| 9 Helpful if someone is hurt, upset or feeling ill |  |  |  |  | **.792/.623** |
| 17 Kind to younger children |  |  |  |  | **.700/.195** |
| 20 Often volunteers to help others (parent, teachers, other children) |  |  |  |  | **.650/.730** |

*^Note.^* ^#p>0.05. Small factor loading estimates were omitted from this table for easy reading; * items are reverse scored (i.e. 0=Certainly True, 2=Not True).^
