# Supplementary material for: The validity of the Strengths and Difficulties Questionnaire (SDQ) for children with ADHD symptoms
Source: PLoS One. 2019 Jun 19;14(6):e0218518. doi: 10.1371/journal.pone.0218518 (PMC6583960; doi:10.1371/journal.pone.0218518)
Supplement: S4 Table — (DOCX) [file pone.0218518.s004.docx]

**S4. Five-factor Item Mapping and Factor Loading of 5-Factor Configural Invariance (Baseline/Follow-up) ESEM Model (Teacher Rating)**

|  | **Factors** | | | | |
| --- | --- | --- | --- | --- | --- |
| **Item** | **‘emotional’** | **‘conduct’** | **‘hyperactivity’** | **‘peer’** | **‘prosocial’** |
| 3 Often complained of headaches, stomach-aches or sickness | **.715/.588** |  |  |  |  |
| 8 Many worries, often seems worried | **.824/.897** |  |  |  |  |
| 13 Often unhappy, down-hearted or tearful | **.698/.708** | .427/ |  |  |  |
| 16 Nervous or clingy in new situations, easily loses confidence | **.849/.756** |  |  |  |  |
| 24 Many fears, easily scared | **.905/.903** |  |  |  |  |
| 5 Often has temper tantrums or hot tempers |  | **.852/.739** |  |  |  |
| 7 Generally obedient, usually does what adults request * |  | **.574/.525** | .435/ |  |  |
| 12 Often fights with other children or bullies them |  | **.905/.922** |  |  |  |
| 18 Often lies or cheats |  | **.787/.733** |  |  |  |
| 22 Steals from home, school or elsewhere |  | **.482/.533** |  |  |  |
| 2 Restless, overactive, cannot stay still for long |  |  | **.894/.920** |  | .442/ |
| 10 Constantly fidgeting or squirming |  |  | **.900/.996** |  | .328/ |
| 15 Easily distracted, concentration wanders |  |  | **.896/.857** |  |  |
| 21 Thinks things out before acting * |  | .401/.363 | **.547/.467** |  |  |
| 25 Sees tasks through to the end, good attention span * |  |  | **.744/.710** |  |  |
| 6 Rather solitary, tends to play alone |  |  |  | **.671/.760** |  |
| 11 Has at least one good friend * |  |  |  | **.562/.656** |  |
| 14 Generally liked by other children * |  | .547/.474 |  | **.565/.471** |  |
| 19 Picked on or bullied by other children |  | .312/ |  | **.576/.699** |  |
| 23 Gets on better with adults than with other children |  |  |  | **.810/.631** |  |
| 1 Considerate of other people’s feelings |  |  |  |  | **.445/.237** |
| 4 Shares readily with other children (treats, toys, pencils etc.) |  |  |  |  | **.115/.081** |
| 9 Helpful if someone is hurt, upset or feeling ill |  |  |  |  | **.685/.453** |
| 17 Kind to younger children |  |  |  |  | **.517/.259** |
| 20 Often volunteers to help others (parent, teachers, other children) |  |  |  |  | **.695/.717** |

*^Note.^* ^* items are reverse scored (i.e. 0=Certainly True, 2=Not True).^
